# Heterodimerization of H3K9 histone methyltransferases G9a and GLP activates methyl reading and writing capabilities

**DOI:** 10.1016/j.jbc.2021.101276

**Published:** 2021-10-05

**Authors:** Nicholas A. Sanchez, Lena M. Kallweit, Michael J. Trnka, Charles L. Clemmer, Bassem Al-Sady

**Affiliations:** 1 Department of Microbiology & Immunology, George Williams Hooper Foundation, University of California San Francisco, San Francisco, California, USA; 2 TETRAD Graduate Program, University of California San Francisco, San Francisco, California, USA; 3 Department of Pharmaceutical Chemistry, University of California San Francisco, San Francisco, California, USA

**Keywords:** chromatin modification, histone methylation, epigenetics, enzyme catalysis, gene silencing, G9a-GLP, heterochromatin, heterodimer, ANK, ankyrin, CLMS, cross-linking mass spectrometry, H3K9, histone 3 lysine 9, H3K9me, histone 3 lysine 9 methylation, MALS, multiangle light scattering, MBP, maltose-binding protein, MLA, methyl-lysine analog, SAM, S-adenosyl methionine, SEC, size-exclusion chromatography

## Abstract

Unique among metazoan repressive histone methyltransferases, G9a and GLP, which chiefly target histone 3 lysine 9 (H3K9), require dimerization for productive H3K9 mono (me1)- and dimethylation (me2) *in vivo*. Intriguingly, even though each enzyme can independently methylate H3K9, the predominant active form *in vivo* is a heterodimer of G9a and GLP. How dimerization influences the central H3K9 methyl binding (“reading”) and deposition (“writing”) activity of G9a and GLP and why heterodimerization is essential *in vivo* remains opaque. Here, we examine the H3K9me “reading” and “writing” activities of defined, recombinantly produced homo- and heterodimers of G9a and GLP. We find that both reading and writing are significantly enhanced in the heterodimer. Compared with the homodimers, the heterodimer has higher recognition of H3K9me2, and a striking ∼10-fold increased turnover rate for nucleosomal substrates under multiple turnover conditions, which is not evident on histone tail peptide substrates. Cross-linking Mass Spectrometry suggests that differences between the homodimers and the unique activity of the heterodimer may be encoded in altered ground state conformations, as each dimer displays different domain contacts. Our results indicate that heterodimerization may be required to relieve autoinhibition of H3K9me reading and chromatin methylation evident in G9a and GLP homodimers. Relieving this inhibition may be particularly important in early differentiation when large tracts of H3K9me2 are typically deposited by G9a-GLP, which may require a more active form of the enzyme.

The animal genome is partitioned into active and inactive regions by gene-repressive structures such as heterochromatin that restrict access to gene-activating factors (1). In early mammalian development, the genome of embryonic stem cells is highly transcriptionally active and features little heterochromatin marked by histone 3 lysine 9 methylation (H3K9me) (2). As differentiation begins, H3K9me heterochromatin expands, remodeling the genome and restricting fate (3). These expansions are carried out by the SETDB1 H3K9 trimethylase (me3) (3, 4) and the G9a and GLP H3K9 mono-and dimethylases (me1/me2). G9a and GLP direct the expansion of large tracks of H3K9me2, which can adopt a lineage-specific pattern (5). This heterochromatin expansion represses lineage inappropriate genes directly or silences enhancers that in turn direct several genes (6).

Like several heterochromatic methyltransferases, G9a and GLP have the capacity to both read and write H3K9me (7, 8, 9, 10). Both enzymes contain an Ankyrin (ANK) repeat domain, which confers methyl-histone binding (reading) activity, and a SET domain, which confers methyltransferase (writer) activity. The presence of methylated substrates appears to stimulate G9a or GLP catalytic activity in an ANK domain-dependent manner (10). Other H3K9 methylases require such positive feedback for either lateral spreading (11, 12) or epigenetic maintenance (13, 14). How the ANK and SET domains of G9a and GLP regulate one another is opaque but may be central to understanding their function in cell fate control.

Unlike other metazoan H3K9 methyltransferases, G9a and GLP must associate with each other to carry out H3K9 methylation (9): When the interaction between the two enzymes is broken *in vivo*, the bulk of H3K9me1 and me2 is lost (15). This is despite the observation that each enzyme is individually capable of methylating histones *in vitro* (8, 9, 10, 16, 17). We hypothesized that forming the G9a-GLP complex (G9a-GLP) has a direct regulatory effect on the H3K9 methylation reaction. However, the biochemical and biophysical nature of homo- and heterotypic associations between G9a and GLP is poorly understood, as are any effects on H3K9me writing and reading that these associations may have.

In this study, we investigated the regulation heterodimerization imposes on G9a and GLP’s ability to read and write H3K9me.

## Results

### G9a and GLP form stable dimers at 1:1 stoichiometry

**Figure 1:**
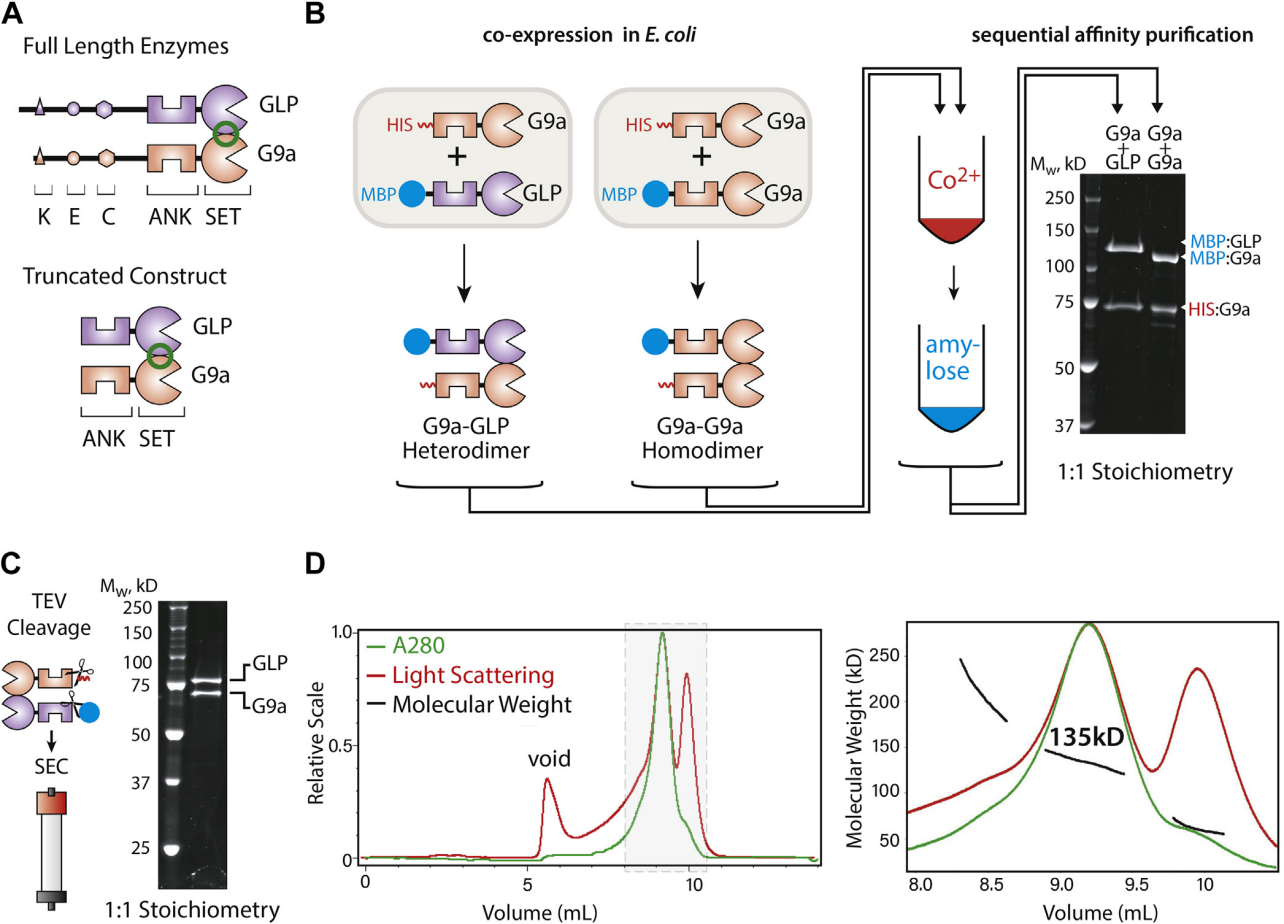
**G9a and GLP form stable 1:1 homo- and heterodimers.***A*, domain architecture of G9a-GLP. *Top*, full-length enzymes. The N-terminus of G9a and GLP feature an automethylation residue (K), an acidic patch (E), and a cysteine-rich region (C). The C-termini contain ankyrin repeats (ANK) that bind H3K9me and a SET domain (SET) which is both the methylation catalytic domain and the dimerization interface (*green circle*). *Bottom*, truncation ANK-SET construct used in this study. *B*, *E. coli* coexpression and purification strategy for identification of G9a homo- and heterodimers. *C*, TEV cleavage of G9a-GLP heterodimers and purification *via* size-exclusion chromatography. *D*, SEC-MALS trace of G9a-GLP. *Left*, full A280 (*green*) and Light Scattering (*Red*) traces. *Right*, magnification of the main peaks with molecular weight determination (*black*). The measured molecular weight of the complex is 135kD (theoretical M_W_ 142kD).

To assay the interplay between reading, writing, and dimerization, we produced G9a and GLP truncated to the C-terminal ANK and SET domains (ANK-SET (10), Fig. 1*A*). To determine if G9a and GLP form homo- or heterodimers, we coexpressed N-terminally 6xHis-tagged G9a (His:G9a) and N-terminally Maltose-Binding Protein (MBP) tagged GLP or G9a (MBP:GLP or MBP:G9a) in *E. coli*. Sequential cobalt and amylose resin purification of either the His:G9a::MBP:G9a homomeric or His:G9a::MBP:GLP heteromeric complexes retains a dimer (Fig. 1*B*). Quantification of SyPRO Red stained bands indicated dimerization at 1:1 stoichiometry for both complexes (Fig. 1*B*). We examined the stability of G9a homo- and G9a-GLP heterodimers using a dilution-based assay, which assesses the off-rate of the complex. Each complex was diluted to 40 nM (about 100 times) and allowed to dissociate for 1 to 2 h. We assessed dimer association by precipitating the His-tagged protein with cobalt resin and determining the fraction of MBP protein that remained bound. We observed little dissociation in either G9a homo or heterodimers (Fig. S1). To exclude the possibility that the N-termini of G9a or GLP counteracts complex formation, we examined complex retention following sequential affinity purification of full-length proteins produced in insect cells. We found them to behave similarly to ANK-SET (Fig. S2, *A* and *B*). Together, these data indicate that G9a forms stable, 1:1 homodimers, as well as heterodimers with GLP.

We next ascertained the number of enzymes per complex in the G9a-GLP heterodimer after removing both the His and MBP tags (Fig. 1*C*). To do so, we determined its molecular weight using size-exclusion chromatography (SEC) followed by multiangle light scattering (MALS). We observed one major peak in our SEC-MALS measurement (Fig. 1*D* left) with a molecular weight of ∼135 kDa (Fig. 1*D*, right), roughly the theoretical molecular weight of our truncated G9a/GLP heterodimer (142 kDa). These data suggest that G9a-GLP is limited to a heterodimeric complex with one G9a and one GLP molecule.

### Heterodimerization facilitates G9a and GLP binding to H3K9 methyl peptides

**Figure 2:**
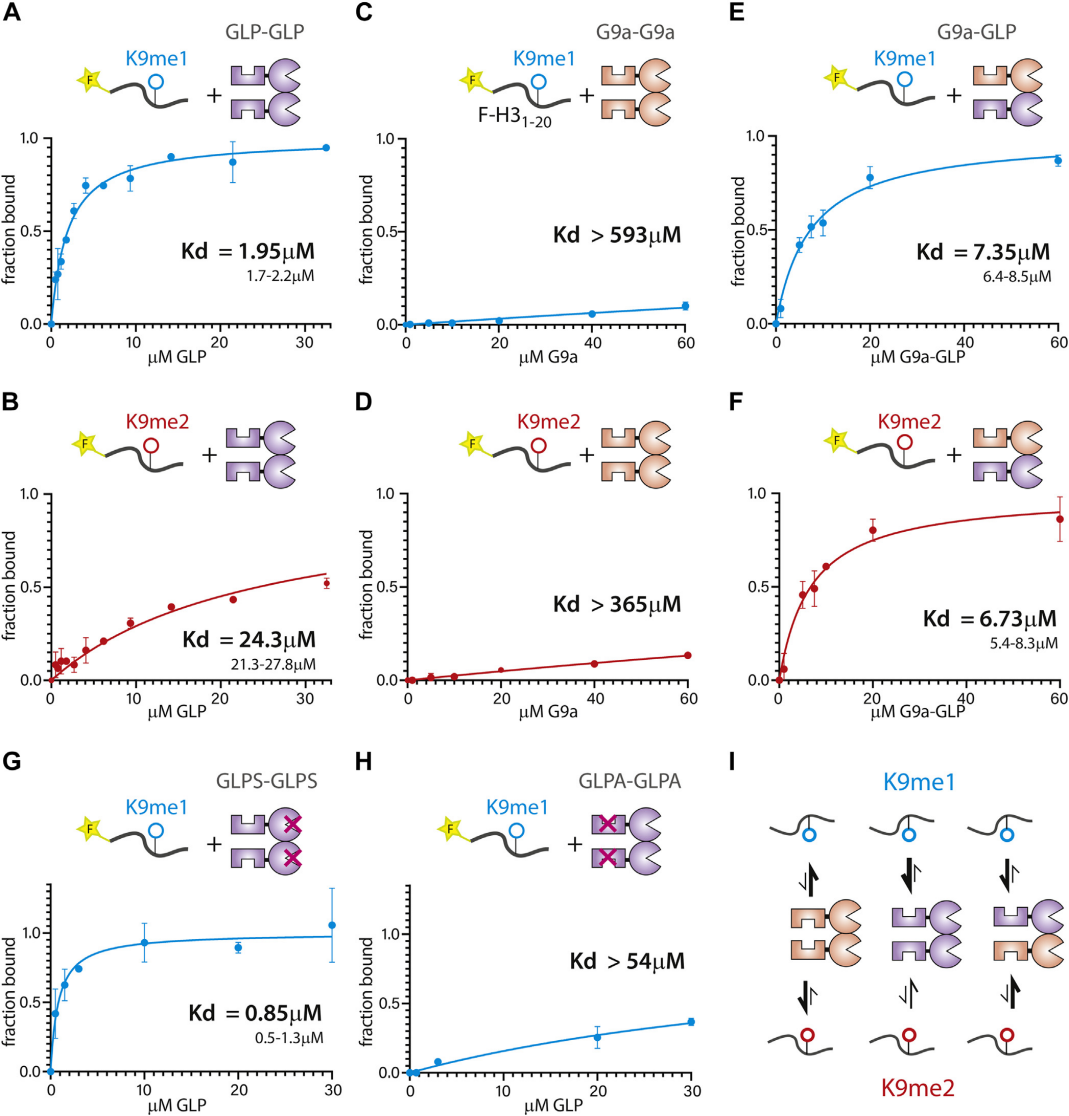
**Heterodimerization facilitates G9a and GLP binding to H3K9 methyl peptides.** Fluorescence Polarization measuring binding of H3K9 mono and dimethyl peptides to *A* and *B*, GLP homodimers; *C* and *D*, G9a homodimers; *E* and *F*, G9a-GLP heterodimers; *G*, GLP homodimer with SET domain catalytic mutation (GLPS); *H*, GLP homodimer with ANK domain mutation (GLPA); K_d_ values are indicated on plots. The 95% confidence interval (CI) is shown for fits with significant binding saturation (*A* and *E*–*G*). For curves with limited saturation and fits with wide range of K_d_ values, the lower bound of the 95% CI is shown. *I*, summary of peptide binding data. Error bars denote standard deviation from independent duplicate experiments.

The presumably monomeric ANK domains of G9a and GLP have been shown to interact with H3K9me1 and H3K9me2 histone tails, to largely similar extents (7, 10). Using fluorescence polarization, we asked whether the ability to engage H3K9me1/me2 is preserved in the dimeric ANK-SET molecule and whether heterodimerization affected this reading function (Fig. 2). Interestingly, except for the high-affinity GLP: me1 association (Fig. 2*A*), the presence of the SET domain in the context of homodimers is broadly inhibitory to H3K9me binding compared with published ANK alone data (7, 10). GLP ANK-SET has 12.5 times reduced binding to me2 peptides (Fig. 2*B*), and we confirmed that H3K9me-binding interactions in the ANK-SET construct are dominated by the ANK domain (Fig. 2, *G* and *H*). Unlike GLP, ANK-SET or full-length G9a has no discernable affinity for me1 or me2 peptides (Figs. 2, *C* and *D* and S2*C*). Heterodimerization restored interaction with me1 and me2 to a range comparable to the ANK domain alone (Fig. 2, *E* and *F*). Taken together, these results suggest that within the ANK-SET homodimeric context, the ANK domains of both G9a and GLP are partially compromised in their ability to bind methyl peptides and that this inhibition is overcome upon heterodimerization (Fig. 2*I*).

### Heterodimerization stimulates G9a and GLP catalytic activity

**Figure 3:**
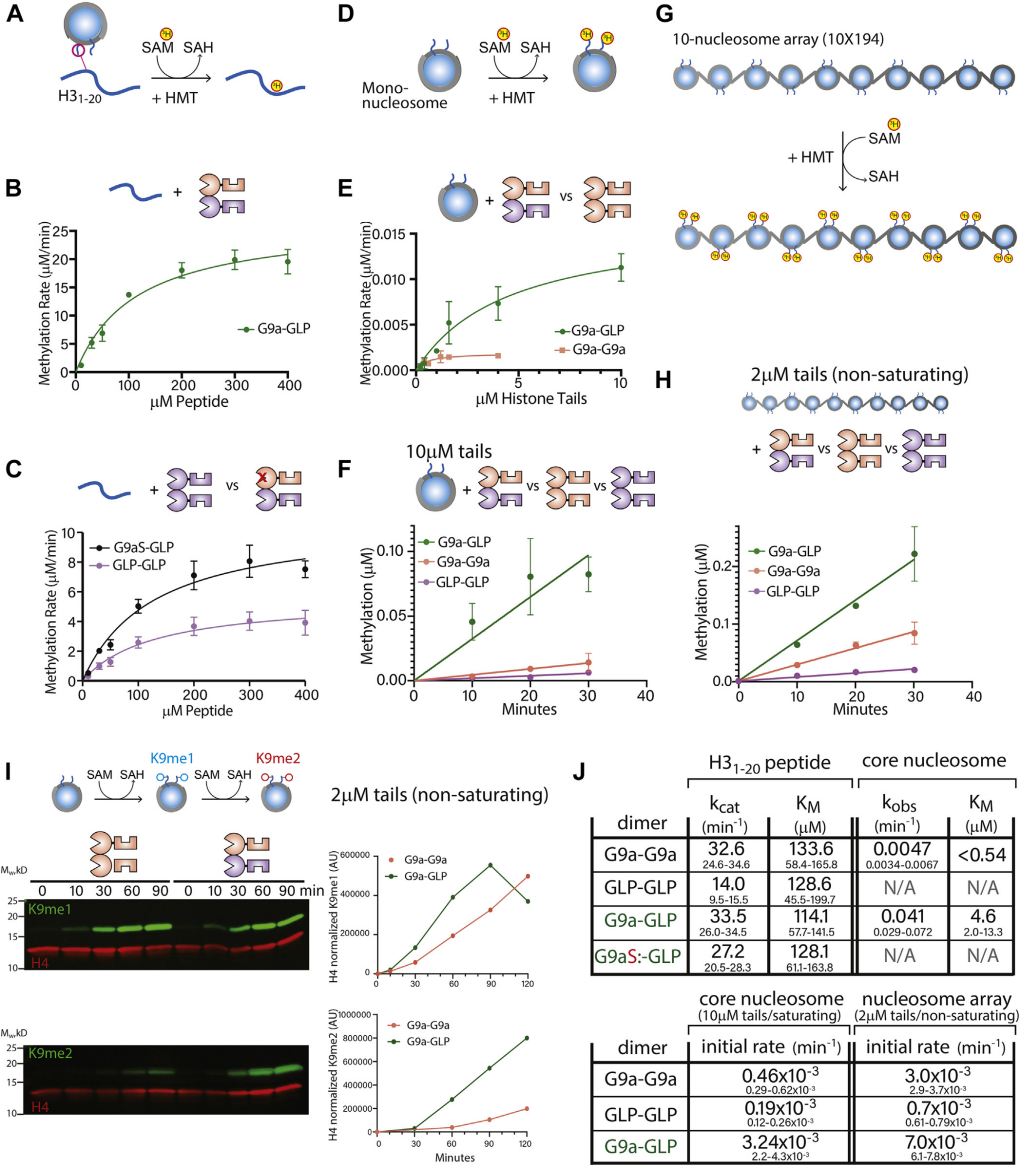
**Heterodimerization stimulates G9a and GLP catalytic activity.***A*, reaction scheme of H3 tail peptide methylation. SAM cofactor was labeled with tritium (^3^H). *B*, Michaelis–Menten fit of G9a-GLP heterodimer methylating H3 tail peptide. *C*, Michaelis–Menten fit of GLP homodimer (*purple*) *versus* G9aS-GLP heterodimer (*black*). *D*, reaction scheme of mononucleosome methylation reactions. *E*, Michaelis–Menten fit of G9a-GLP heterodimer (*green*) and G9a homodimer (*pink*) methylating mononucleosomes. *F*, initial rate comparison of G9a-GLP (*green*), G9a homodimer (*pink*), and GLP homodimer (*purple*) methylating mononucleosomes under saturating conditions (10 μM histone tails). *G*, reaction scheme of nucleosome array methylation reactions. *H*, initial rate comparison of G9a-GLP (*green*), G9a homodimer (*pink*), and GLP homodimer (*purple*) methylating 10-nucleosome arrays under nonsaturating conditions (2 μM histone tails). *I*, *left*, Western blot time course of H3K9me1 or H3K9me2 (*green*) production on mononucleosomes under nonsaturating conditions (2 μM histone tails). H4 was blotted for as an internal control (*red*). *Right*, the H4-normalized production of H3K9me1 or me2 by G9a (*pink*) or G9a-GLP (*green*) is plotted over time. *J*, kinetic parameters for histone peptide and nucleosome methylation. Values reflect kinetic parameters (*upper value*) and 95% confidence intervals (*lower values*). k_obs_ for nucleosomes denotes a first-order rate constant under conditions where the SAM pocket is not saturated (see Fig. S3, *A* and *B*). Error bars denote standard deviation from independent duplicate experiments.

We next asked whether dimerization affects the methyltransferase activity of G9a-GLP. We determined Michaelis–Menten kinetic parameters of G9a and GLP homodimers as well as the G9a-GLP heterodimer using an H3 histone tail peptide substrate (H3_1–20_ (11), Fig. 3, *A*–*C* and *J*). While the K_M_ of all enzyme species was similar (Fig. 3*J*), we observed changes of the k_cat_ within the heterodimer beyond those expected from equal contributions from G9a and GLP: The G9a and GLP k_cats_ are ∼33 min^−1^ and ∼14 min^−1^, respectively. Rather than an intermediate value, G9a-GLP’s k_cat_ is ∼33 min^−1^, suggesting that one or both enzymes are more active in the heterodimer than their respective homodimers. To determine which enzyme is stimulated in the heterodimer, we made point mutations in either G9a (G9aS, Y1120V, Y1207F) or GLP (GLPS, Y1240F) abrogating their catalytic activity while still allowing dimerization with a wild-type allele of their binding partner (15, 18). Comparing G9aS-GLP to GLP-GLP, we observe that GLP’s k_cat_ increased approximately two times upon heterodimerization (Fig. 3*C*). Activity comparisons of G9a-GLPS and G9a-G9a suggested a very minor increase in activity of G9a in the heterodimer (Fig. S4*C*), indicating that only GLP’s activity is meaningfully enhanced in the heterodimer. Thus, these data imply a modest catalytic enhancement in G9a-GLP *versus* the homodimeric forms.

Next, we asked if the G9a-GLP heterodimer exhibits altered methylation kinetics on nucleosomes (Fig. 3*D*). We first measured Michaelis–Menten parameters under multiple turnover conditions, just like for H3_1–20_, for G9a-GLP, and G9a-G9a on a reconstituted mononucleosome. In striking contrast to our observations on H3_1–20_, G9a-GLP’s k_obs_ is ten times higher than that of G9a-G9a (Figs. 3, *E* and *J* and S4*B*), which we confirmed measuring initial rates under near-saturating conditions. Under these conditions, the heterodimer also exhibits higher activity (approximately eight times higher k_obs_) than G9a-G9a, with the GLP homodimer the slowest of the three enzyme constructs (Fig. 3*F*). We repeated this initial rate comparison for 10-nucleosome arrays (Fig. 3*G*), to test whether this enhancement holds for chromatin substrates. However, due to technical limitations, we could only do so under nonsaturating conditions for G9a-GLP (2 μM histone tails). Nevertheless, even in this condition, G9a-GLP was the fastest of the three enzymes, and GLP the slowest (Fig. 3*H*). Surprisingly, while G9a-GLP accelerates H3K9me1 and me2 production on nucleosomes *versus* G9a, the overall rate enhancement appears to be coded most strongly in the H3K9me1-H3K9me2 transition (Fig. 3*I*). We conclude that chromatin substrates bring to fore the intrinsic catalytic differences between homo and heterodimers, revealing a dramatically increased k_obs_ for G9a-GLP. We note that concomitant with this k_obs_ increase, however, we also noticed an increase in the nucleosome K_M_ for G9a-GLP (Fig. 3, *E* and *J*). Such joint increases or decreases in k_cat_ and K_M_ on chromatin substrates can be indicative of changes in the number of substate encounter modes compared with simpler substrates (11).

### The heterodimer interacts with chromatin in a G9a-specific manner, but unlike G9a, recognizes methylated nucleosomes

**Figure 4:**
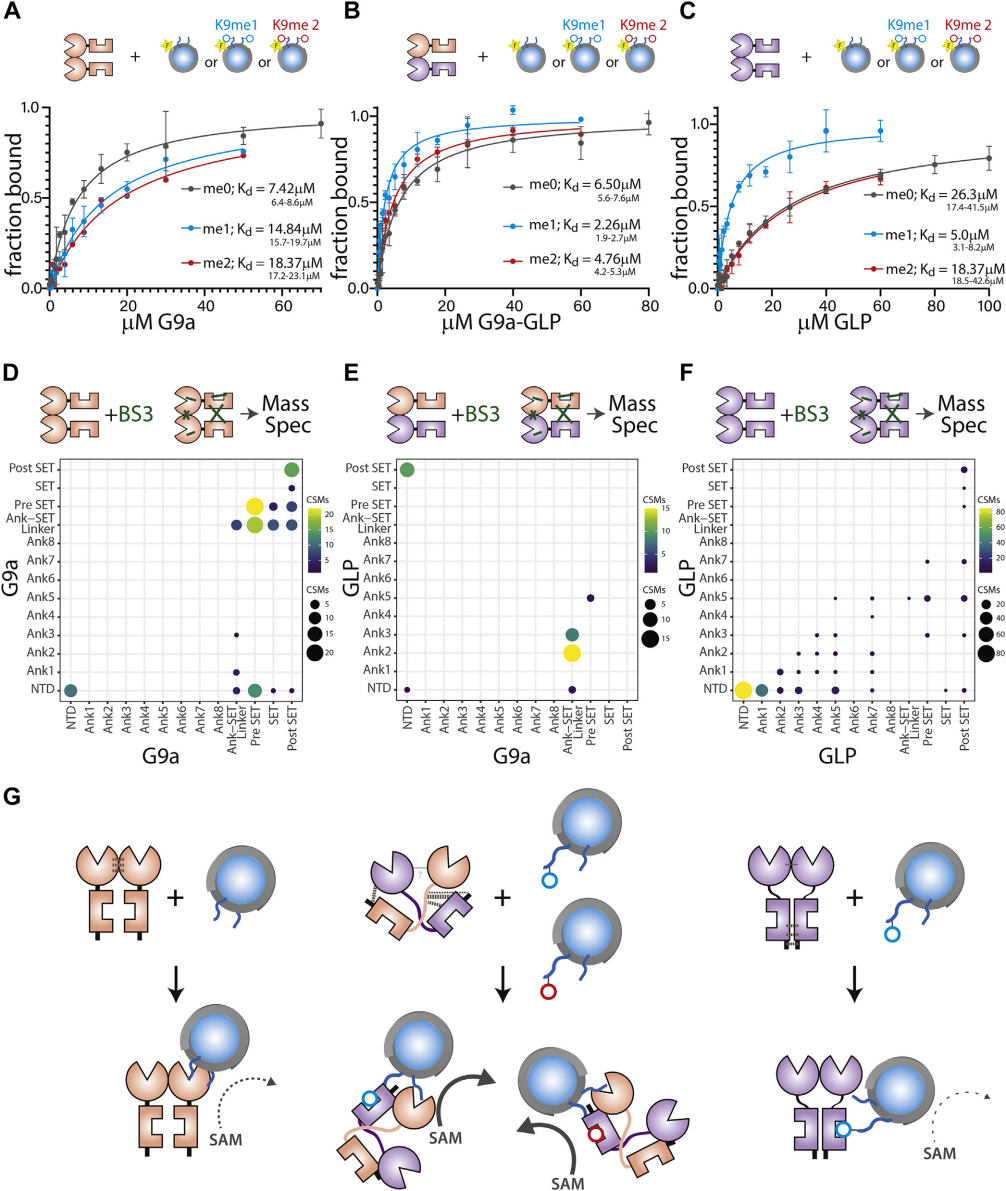
**Unique nucleosomes engagement and ground statement conformation of G9a-GLP heterodimer.***A*–*C*, fluorescence polarization of unmethylated (H3K9me0; *grey*), H3K9me1 monomethylated (*teal*), and H3K9me2 dimethylated (*red*) mononucleosomes containing 147 bp “601” DNA 5ʹ fluorescein-labeled bound to (*A*) G9a (*B*) G9a-GLP (*C*) GLP-GLP. The 95% confidence interval (CI) is shown for all K_d_s. *D*–*F*, cross-linking Mass Spectrometry for G9a (*D*), G9a-GLP (*E*), and GLP (*F*). *Dot* size and color indicate number of cross-linked spectral matches (CSM) summarized for domain pairs. Note that G9a and GLP data by necessity include intra- and interprotomer cross-links. *G*, model. G9a protomers interact predominantly *via* the SET domain. The ANK domains are in an auto-inhibited state. Nucleosome engagement is *via* the H3 tail. G9a-GLP makes cross-protomer N- to C-terminal contacts, leading to a different ground state conformation with the ANK domains free. G9a and GLP (shown) ANK could engage with H3K9me1 or me2 nucleosomes. The engagement mode of G9a-GLP with the nucleosome results in increased catalysis. GLP associates strongly at the N-terminus, allowing the ANK domains to access methylated residues. However, GLP has limited engagement with the nucleosome *via* the SET domain, resulting in slow catalysis.

As in the above studies with the H3 tail peptide, we used fluorescence polarization to measure the binding of each ANK-SET dimer on unmethylated or H3K9 mono -or dimethylated mononucleosomes. On the unmethylated nucleosome, for G9a-G9a and G9a-GLP homo- and heterodimers, respectively, we measure a similar K_d_ in the range of ∼6 to 7 μM (Fig. 4, *A* and *C*). For G9a, this interaction requires a catalytically active SET domain (Fig. S5*A*). GLP-GLP exhibits a significantly lower affinity, with a K_d_ of 26.3 μM (Fig. 4*B*), indicating that binding to the unmethylated nucleosome in the G9a-GLP heterodimer is mostly driven by G9a. The binding of unmethylated nucleosomes by the ANK-SET of G9a-GLP does not appear to depend on contacts with DNA, unlike for other HMTs (11, 12), as it does not show appreciable binding to DNA compared with a Clr4 HMT control (Fig. S6). Thus, the engagement mode of G9a and G9a-GLP with unmethylated nucleosomes seems to require interaction with the H3 tail *via* the SET domain active site. The loss of engagement with the nucleosome in G9aS Y1120V/Y1207F may be due to more extensive rearrangements in the G9a SET domain H3 binding pocket mutants. Even though the data points to the SET domain–H3 tail contact as necessary for nucleosome engagement, given the increased affinity for nucleosome over H3 peptides (see Fig. S2, *C* and *D*), G9a/G9a-GLP likely make additional contacts to the histone octamer.

We also assessed how H3K9 mono- and dimethylation impact the association of G9a, GLP, or G9a-GLP to nucleosomes. The affinity of G9a to H3K9me1 and me2 nucleosomes decreases compared with unmethylated nucleosomes (Fig. 4*A*). This result is consistent with the G9a SET catalytic domain making the primary nucleosome interactions, as 1. The SET binds methylated tails much more weakly than the ANK domain, as 2. The G9a ANK domain in the ANK-SET context does not recognize H3K9me1/2 peptides (Fig. 2, *C*, *D*, *G* and *H*). GLP binds relatively tightly to H3K9me1, but not H3K9me2 nucleosomes (Fig. 4*B*). Since GLP binds H3K9me1 peptides tightly, this may suggest that GLP can engage with the nucleosome *via* ANK domains when H3K9 is monomethylated, but only weakly *via* the SET domain in the unmethylated state. In contrast, the G9a-GLP heterodimer exhibits mild enhancement of binding H3K9me1 and me2 nucleosomes over the unmethylated state. However, since fluorescence polarization cannot distinguish the engagement mode of G9a-GLP, we cannot conclude to what degree ANK and SET engagement contributes to these overall K_d_s. Nevertheless, only G9a-GLP can recognize both H3K9me1 and H3K9me2 on nucleosomes.

### G9a, GLP, and G9a-GLP exhibit unique inter- and intramolecular contacts

The unique catalytic and substrate-binding behaviors of G9a-GLP may be encoded by a different ground state conformation for the heterodimer or by induced changes that occur upon substrate engagement and that are not evident in the ground state. To distinguish between these possibilities, we conducted BS3 cross-linking followed by Mass Spectrometry of G9a and GLP homodimers and G9a-GLP heterodimer. We note that we may not be able to detect all protein–protein contacts *via* this method (see Supporting discussion). Nonetheless, we observed marked differences between all three dimers. Cross-links in G9a are clustered in the SET domain, most prominently in the “pre-SET” and “post-SET” regions (Fig. 4*D*). While GLP also displayed contacts in the SET domain, it displayed much more prominent cross-links at the N-terminus of the ANK-SET construct, upstream of the ANK (NTD, Fig. 4*F*). We note that for both G9a and GLP, we cannot differentiate whether these contacts are intra- or intersubunit. However, we can infer that the most frequent contacts are largely within the same region of the protein, N terminus/ANK *versus* C terminus (ANK-SET linker and pre-SET, SET, and post-SET). For G9a-GLP, we can isolate intersubunit cross-links, as we can filter out intrasubunits cross-links, which results in overall isolating fewer cross-links compared with the homomeric dimers. Surprisingly, we find a different pattern of cross-links for G9a-GLP, where the strongest cross-links are from the N-terminus of G9a to the post-SET of GLP or from the ANK of GLP to the ANK-SET linker or pre-SET of G9a. This is qualitatively different from G9a or GLP, pointing to a different ground state conformation. A repeat of this experiment with the MBP-tagged proteins yielded a similar overall picture (Fig. S7), though in this case, we uncovered several MBP cross-links, which may have biased the overall distribution of captured contacts.

## Discussion

Unusual among metazoan histone methyltransferases, G9a and GLP form dimers *in vivo*, and this dimerization is essential for function (9). We discuss our findings regarding the ability of these proteins to (1) homo-and heterodimerize and the unanticipated consequences of heterodimerization on both H3K9me, (2) reading, and (3) writing.

### Constitutive dimerization

The degree to which dimerization, or the stability of dimers, is regulated by homo- and heteromeric associations is unknown. Our data argue that the ANK-SET portion of G9a and GLP homo- and heteromeric species form 1:1 dimers with low apparent dissociation and that efficient dimerization is not limited in the homomeric context (Figs. 1 and S1). However, *in vivo* experiments indicate that when G9a and GLP are present at equal cellular stoichiometry, the heterodimer is the preferred form and that homodimers can form when G9a or GLP is in excess (9). This *in vivo* preference may be due to regulation outside the ANK-SET domain. Taken together with our results, a picture emerges where G9a and GLP constitutively form homo- or heterodimers with relative pools determined by the steady-state accumulation of either protein. Homodimers, when formed, may then execute unique functions, for example, in DNA repair for GLP (19), and in terminal muscle differentiation, where G9a and GLP control nonoverlapping genes sets (20). We speculate that the heterodimer, with increased catalysis on chromatin and product recognition (below), is required when large domains of H3K9me2 are first formed and then maintained through cell division (5, 6), and that specialized and local chromatin methylation or methylation of nonhistone targets, may be carried out by homodimers.

### Deinhibition of H3K9me2 reading

Our data suggest that H3K9me reading, in particular, H3K9me2 is inhibited within the ANK-SET homodimer. Only the heterodimer appears capable of strong H3K9me2 recognition (Fig. 2), while both GLP-GLP and G9a-GLP can recognize H3K9me1. This differs from findings on the presumably monomeric G9a and GLP ANK domains alone as measured by fluorescence polarization (7) or ITC (10). Because the affinities we measure for H3K9me1/2 binding, where observed, are within twofold of these published affinities for ANK alone (7, 10), we do not believe that the ANK domains in our hands have lower specific activity. Instead, we interpret our data to indicate that either (1) inhibitory contacts between ANK and SET or ANK and ANK across the two dimers are alleviated in G9a-GLP or (2) heterodimerization induces a conformational change within the ANK. Our cross-linking mass spectrometry (CLMS) results favor differences in the ground state conformation of the heterodimer, which may “liberate” the ANK domains of G9a and possibly GLP to engage with H3K9me (Fig. 4, *D*–*F*, model Fig. 4*G*). Beyond these interactions in the ground state where the protein has not engaged substrate, H3K9me ANK-binding sites may be initially unavailable in the naïve protein but become fully available following initial nucleosome engagement to bind adjacent H3K9me sites. This could account for the observation of H3K9me1 or me2-specific stimulation of methylation by G9a and GLP homodimers on chromatin circles (10).

### Stimulation of chromatin methylation

We observe both modest, constitutive activation of GLP on H3 tail peptides and a dramatic increase in the k_obs_ toward nucleosomes compared with the homodimers. We believe two mechanisms can account for these results:

#### Relief of general autoinhibition

Like other histone methyltransferases, G9a and GLP may be limited by autoinhibition. The fission yeast Clr4 enzyme contains an autoinhibitory loop that is relieved by automethylation (21), and similar inhibitory loops have been shown for PRC2 and NSD1 (22, 23). The constitutive activation we observed in GLP upon heterodimerization (Fig. 3) may be due to the restructuring of an autoinhibitory loop in the post-SET domain (24). Additionally, the inability of G9a to recognize H3K9 methylation on peptides or nucleosomes (Figs. 2 and 4) suggests that the ANK of G9a is in an inaccessible state.

#### Optimized modes of substate engagement

The observation that GLP binds poorly to nucleosomes indicates that G9a and G9a-GLP feature more optimal nucleosome engagement specifically, although this is not the case for the peptide, as the K_M_s values of all three enzymes are similar. Therefore, while engagement of the SET domain with the H3 peptide is critical for nucleosome binding (Fig. S5*A*), additional contacts are likely made by G9a/G9a-GLP to other surfaces of the histone octamer. Further, G9a-GLP likely features a different ground state conformation that may enable more productive engagement with the nucleosome compared with G9a, resulting in an increased k_obs_. Given that G9a′s K_M_ is significantly lower than its nucleosomal K_d_ (unlike in the G9a-GLP case), this inefficient nucleosome catalysis could be explained by either an increase in nonproductive binding modes (11) or inefficient product release (25). While we cannot determine the true k_cat_ for nucleosomes given signal limitation (see Experimental procedures), we note that the specificity constant (k_cat_/K_M_) is not raised compared with peptides, at least on mononucleosomes. However, the elevated k_obs_ observed on nucleosome arrays compared with mononucleosomes, even at subsaturating conditions (Fig. 3*J*), hints that G9a-GLP’s true k_cat_ on chromatin may still be higher.

The unique ability of the G9a-GLP molecule to recognize H3K9me2 as well as methylate chromatin, especially compared with the G9a homodimer (Fig. 4*G*), begins to explain the strong requirement of the heterodimer for global maintenance of H3K9me2 and normal embryogenesis.

## Experimental procedures

### Purification of G9a/GLP homo- and heterodimers

To isolate the ANK-SET (10) G9a-GLP heterodimer, we coexpressed N-terminally tagged His:G9a and MBP:GLP from a single plasmid (QB3 Berkeley Macrolab expression vectors) in *E. coli* DE3 Rosetta cells and performed sequential cobalt- and amylose-charged resin affinity chromatography purification. Lysis, purification, and tag cleavage were broadly as described (26) with modifications (see Supporting methods for details).

### Size-exclusion chromatography coupled to multiangle light scattering

For size exclusion, protein samples were injected at 10 μM into a silica gel KW804 chromatography column (Shodex). For MALS, the chromatography system was coupled to an 18-angle light-scattering detector (DAWN HELEOS II, Wyatt Technology) and a differential refractometer (Optilab-rEX, Wyatt Technology). Data collection was done at a flow rate of 0.4 ml per minute. SEC-MALS data were collected and analyzed using Astra 7 software (Wyatt technology).

### Preparation of mononucleosome substrates

Histone proteins and nucleosomes were purified as described (11, 26) with modifications (see Supporting methods). For fluorescence polarization, nucleosomes were reconstituted with a 5ʹ fluoresceinated DNA template. H3K9 dimethylation was installed *via* Methyl-Lysine Analog (MLA) technology as described (10, 26). K9 MLA-monomethylated H3 was purchased from Active Motif. H3K27A was introduced by site-directed mutagenesis and purified as described (26).

### Methylation assays

Substrate peptides or nucleosomes were mixed in a solution containing 9 μM tritiated S-Adenosyl Methionine (SAM, PerkinElmer) cofactor, and reactions were initiated upon addition of 0.4 to 0.8 μM enzyme. Peptide reactions were supplemented with 1000 μM cold SAM to fully saturate the SAM-binding pocket (Fig. S3). Due to signal limitations, nucleosome reactions were not supplemented with SAM (see Supporting methods). Reactions were run in 100 mM Tris pH 8, 100 mM KCl, 1 mM MgCl_2_, 20 μM ZnSO_4_, 10 mM BME and quenched with laemmli buffer. Peptide reactions were performed as described (11). Nucleosome methylation reactions were read out *via* autoradiography as described (11), except that proteins were separated on an 18% SDS PAGE gel.

### Kinetics

All methylation reactions (except Fig. S4*A*) were performed under Multiple Turnover conditions (25). To determine initial rates, methylation time courses were traced at various concentrations of H3K9-containing substrate while keeping the concentration of SAM and enzyme constant. Plots of initial rate *versus* concentration H3K9 substrate measured in duplicate or triplicate were fit to V = Vmax∗[S]/(K_M_ + [S]) using Prism software to extract K_M_ and k_cat_ (or k_obs_) values as well as the 95% confidence interval bounds of the fit. See Supporting methods for special considerations for nucleosome kinetics.

### Fluorescence polarization

Polarization assays with fluoresceinated H3_1–20_ K9me1 or K9me2 peptides, 147 bp 601 DNA template, and core nucleosomes were performed and K_d_ values estimated as described (11) with the following modifications: The reaction buffer was 100 mM Tris pH 8, 100 mM KCl, 1 mM MgCl_2_, 20 μM ZnSO_4_, 10 mM BME, 0.1% NP-40. The concentration of peptides and nucleosomes was 150 nM and 200 nM for DNA. Polarization measurements were conducted on a Biotek Cytation 5 (peptide and nucleosome) or Molecular Devices Spectramax (DNA) plate reader with low-volume plates (Corning) in either 10 μl (peptide, nucleosome) or 40 μl (DNA) total volume.

### Cross-linking Mass Spectrometry (CLMS)

GLP, G9a, or G9a-GLP was either cleaved from His/MBP tags (Fig. 4) or left uncleaved (Fig. S7, see Fig. S8 for tag and domain annotations) and dialyzed into cross-linking buffer (25 mM HEPES pH 7.5, 140 mM KCl, 0.5 mM MgCl_2_, 1 mM BME). CLMS was generally performed as described (27). Detailed methods and measurement parameters can be found in Supporting methods.

## Data availability

Thermo RAW files are deposited in the MassIVE repository (https://massive.ucsd.edu) with accession: MSV000088143.

Annotated peaklists supporting the cross-linked peptide assignments reported in Table S1 can be viewed online at the MS-Viewer website (https://msviewer.ucsf.edu/prospector/cgi-bin/msform.cgi?form=msviewer) with the following Search Keys:

G9a-G9a: bx6qcjbsh8

G9a-GLP: gle6gczo0a

GLP-GLP: rjczqcqrjj

## Supporting information

This article contains supporting information (9, 11, 15, 27).

## Conflicts of interest

The authors declare that they have no conflicts of interest with the contents of this article.

## Supporting information

Supporting Methods, Discussion and Figures S1–S8
