# Supplementary material for: Heterodimerization of H3K9 histone methyltransferases G9a and GLP activates methyl reading and writing capabilities
Source: J Biol Chem. 2021 Oct 5;297(5):101276. doi: 10.1016/j.jbc.2021.101276 (PMC8564726; doi:10.1016/j.jbc.2021.101276)
Supplement: Supporting Methods, Discussion and Figures S1–S8 [file mmc1.pdf]

Supporting material:

Supporting Methods

Detailed E. coli protein expression, purification, and tag cleavage methods
Cells co-expressing His and MBP constructs were lysed on ice via sonication in lysis buffer (100mM Tris pH 8, 300mM NaCl, 10% glycerol (v/v), 0.1% Tween-20, with freshly added 1mM β-mercaptoethanol (BME), 1mM PMSF, 5mM Benzamidine, 200µM Leupeptin, Aproptinin, Pepstatin, Phenantroline). The clarified lysate was then bound to cobalt-charged resin (Takara) for 1hr and washed twice with lysis buffer. His tagged proteins were eluted with lysis buffer containing 400mM imidazole and bound immediately to amylose resin (NEB) for 1hr. MBP tagged proteins were eluted with lysis buffer + 20mM maltose. Affinity tags were then removed by incubation with 12mg TEV protease for 1hr at 25°C. TEV protease was absorbed to cobalt resin and the cleaved heterodimer was further purified by size exclusion chromatography (Superdex 200 Increase 10/300 column) and buffer exchanged into storage buffer (100mM Tris pH 8, 100mM KCl, 10% glycerol, 1mM $MgCl_2$, 20µM $ZnSO_4$, 10mM BME). Homodimeric MBP:G9a or MBP:GLP ANK-SET constructs were purified as above, omitting the cobalt resin purification. All protein constructs were quantified using SDS page with BSA standards and Sypro Red stain.

Detailed chromatin preparation methods
Histone octamers were assembled on either 147bp '601' positioning sequence DNA or an array of 10 repeats of '601' positioning sequence with 47bp flanking DNA (derived from the 12-repeat array in (11)). Following assembly, nucleosomes were dialyzed overnight into storage buffer (above) or FP storage buffer (50mM HEPES pH 7.5, 100mM KCl, 10% glycerol) depending on the application. Dialyzed samples were concentrated using 10kda Millipore Sigma Amicon Ultra Centrifugal Filter Units.

Dilution experiment
Purified His:G9a::MBP:GLP or His:G9a::MBP:G9a complexes were diluted to 40nM in lysis buffer and allowed to dissociate for 1-2hr at room temperature in a volume of 20µL. 7µL cobalt-charged resin was added to each sample and incubated for 30min to allow resin binding. The resin was washed twice with lysis buffer and 20µL 1x Laemmli Buffer was then added to the resin. The ratio of MBP tagged protein to His tagged protein was assessed via SDS page with BSA standards and Sypro Red stain. Fraction assembled was =1 for a "stock protein" (SP) control that was not put through this assay and had a concentration of >4µM.

Insect Cell protein expression and purification
Baculovirus containing full-length His:G9a and STREP:GLP co-expression cassettes (QB3 Berkeley Macrolab) were used to infect Sf9 cells (Expression Systems, Davis, California) grown in ESF921 media. Cells were infected for 72hr at an MOI of 0.1. Infected cells were flash-frozen and stored at -80°C until thawed for purification. Cells were lysed on ice via sonication in lysis buffer (100mM Tris pH 8, 300mM NaCl, 10% glycerol (v/v), 0.1% Tween-20, with freshly added 1mM β-mercaptoethanol (BME), 1mM PMSF, 5mM Benzamidine, 200µM Leupeptin, Aproptinin, Pepstatin, Phenantroline). The clarified lysate was then bound to streptactin superflow resin (IBA) for 1hr and washed twice with 100mM Tris pH 8, 750mM NaCl, 10% glycerol (v/v), 0.1% Tween-20, freshly added 1mM BME. Strep-tagged proteins were eluted with lysis buffer containing 5mM desthiobiotin and bound immediately to cobalt-charged resin (Takara) for 1hr. His tagged proteins were eluted with lysis buffer + 400mM imidazole. Complex formation was assessed via western blot.

Electromobility Shift Assay
To assess binding to 147bp '601' DNA via EMSA, G9a-GLP or Clr4 were incubated with 200nM of a 147bp 601 DNA template in 50mM HEPES pH 7.5, 100mM KCl, 10% glycerol, 0.1% NP-40 in a 10µL volume. Upon mixing, 50% glycerol was added to 5% final and samples were run on a 5% Tris-Glycine Native Gel. Samples were imaged using Chemidoc MP Imaging System (Biorad).

Expression in E. coli and purification of full-length G9a and SET domain G9a-GLP heterodimer
The purification strategy for SET domain G9a-GLP heterodimer was as for the ANK-SET version, with identical purification affinity tags. Full-length human G9a was cloned with an N-terminal MBP tag (MBP:FL-G9a), analogous to ANK-SET MBP:GLP, and purified as above, without cleavage of the MBP tag to preserve maximal yield.

Enzyme kinetics with nucleosomes
For peptide reactions, we saturated the SAM binding pocket (see main methods) with a mixture of tritiated and non-tritiated SAM to measure kinetic parameters under pseudo-first-order conditions. For nucleosome experiments due to signal limitations, we did not add non-tritiated SAM, thus, we could not fully saturate the SAM pocket. Fully saturating the SAM pockets requires the addition of ~100 times more non-tritiated than tritiated SAM, reducing measurable signal. As the enzymes have lower turnover on nucleosomes than peptides, we lose signal at low nucleosome concentrations if we attempt to saturate the SAM pocket. Hence we report k_{obs} values for nucleosomes. We show that measured nucleosome k_{obs} values are indeed limited by the available SAM (Supporting Figure 3B).

Cross-linking Mass Spectrometry (CLMS)
Minor modifications from reference (27) and detailed methods: Crosslinking was performed at 4μM monomer and 0.75mM BS3 crosslinker for 33min at room temperature. The reaction was quenched with Tris HCl pH 7.5. Crosslinking was confirmed via SDS-PAGE. Total protein was precipitated with cold acetone. Size exclusion fractions enriched in crosslinked peptides were combined into two mass spectrometry samples and each one was acquired over a 90-minute UPLC gradient acquiring sequential HCD and EThcD product ion spectra on a Fusion Lumos mass spectrometer (Thermo Scientific). Two LC-MS acquisitions, corresponding to the two SEC fractions, were performed for each of the three dimeric samples: G9a-G9a, GLP-G9a, GLP-GLP. Peaklists were generated using PAVA (UCSF) for each dissociation method and searched with Protein Prospector 6.3.23. The search database included sequences for human G9a and GLP methyltransferases, as well as four contaminating proteins detected in moderate abundance (from *E. coli* chaperones and bovine protein standards). A randomized fasta database that was 10x longer than the target database was used for FDR estimation. The database search used trypsin specificity with up to 2 missed cleavages and the precursor and product ion tolerance were set at 15 ppm and 20 ppm respectively. Carbamidomethylation of Cys was used as the constant modification while oxidation of Methionine and "dead-end" modification of Lys and the protein N-terminus by DSS/BS3 as variable modifications. DSS/BS3 was specified as the crosslinking reagent with a mass modification range between 778-4378 Da. Up to 3 variable modifications per peptide were allowed.

Only sample-specific protein crosslinks are reported. Crosslinked spectral matches (CSMs) were classified by picking an SVM.score threshold corresponding to a 1% false discovery rate (FDR). CSMs are reported at the unique-residue-pair level in Supplemental Table 1 and plotted at the domain-pair level in Figure 4. Domain pair reporting was done by counting the number of product ion spectra above the SVM.score threshold (eg CSMs) that mapped to each domain pair. CLMS classification and reporting were performed using Touchstone, an in-house R library.

Supporting discussion on Crosslinking Mass Spectrometry of G9a, GLP, and G9a-GLP

In this experiment, we crosslinked all three dimers with the BS3 crosslinker. One surprising finding for G9a-GLP is the absence of specific G9a-GLP crosslinks via the SET domain. Mutations in the SET domain have been shown to disrupt heterodimers *in vivo* (15) and in HEK cells, SET domains of G9a and GLP have been shown to co-immunoprecipitate (9). It is possible we did not observe crosslinks at these sites for the following technical reasons: 1. For G9a-GLP, crosslinks are excluded that occur over identical sequences in both proteins, as those cannot be assigned. For example, G9a and GLP share the cross-linkable sequence CWYDKDGR in the pre-SET domain. Other short identical sequences exist that would not be assigned should those result in crosslinks. The CLMS scoring algorithm prioritizes intra-protein results over analogous inter-protein results so CSMs matching these peptide would be reported as either G9a-G9a or GLP-GLP crosslinks. 2. It is possible that our crosslinking was not complete. We chose a concentration of BS3 crosslinker (0.75mM) that results trapping of a dimeric form and some minor accumulation higher molecular weight forms, but not significant accumulation of aggregates. Under these conditions, the monomeric form is not fully depleted. Hence it is possible that potential contacts did not get trapped by crosslinking at this BS3 concentration. 3. It is possible we did not identify a particular crosslink because the physiochemical properties of the crosslinked peptides result in poor ionization or chromatography such that the precursor ion is not selected for MS2 in a complex mixture.

Supporting Figure 1: Stability of G9a-G9a and G9a-GLP complexes through a dilution assay. A. Experiment scheme. His:G9a coexpressed with either MBP:G9a or MBP:GLP was purified by sequential affinity purification as in Figure 1. The complexes were diluted and kept at 25°C for 1-2hrs. After this incubation, His:G9a was isolated via cobalt resin precipitation. **B.** The relative amount of His and MBP tagged proteins retained after cobalt resin precipitation was quantified by SyPRO Red staining and normalized to a stock protein (SP, > 4μM) of purified undiluted protein (fraction associated). The highest dilution for G9a-G9a and G9a-GLP, 40nM or > 100 times dilution, is shown.

Supporting Figure 2: Behavior of Full-length G9a and GLP. A. Full-length GLP and G9a were N-terminally tagged with a STREP or 6XHis tag, respectively, co-expressed from a baculoviral construct in Sf9 insect cells, and isolated by sequential STREPTACTIN (Tactin) and Cobalt (Co^{2+}) affinity resins. **B.** Western blots of baculovirus-infected or uninfected Sf9 lysates (LEFT) or affinity resin eluates (RIGHT) with anti-His tag or anti-STREP tag antisera. The single-channel and merged images are shown. **C.** Fluorescence polarization with *E. coli* expressed MBP:FL-G9a. The estimated (due to lack of saturation) lower limit of the K_d for H3K9me0 is shown, the K_d for H3K9me2 could not be determined. **D.** Fluorescence polarization with ANK-SET G9a and H3K9me0. The estimated (due to lack of saturation) lower limit of the K_d is shown. Error bars in C. and D. indicate standard deviation of two repeats.

Supporting Figure 3: S-adenosyl methionine K_M and salt effects on the peptide K_M A. LEFT: The K_M for SAM was determined with G9a-GLP and $H3_{1\text{-}20}$ peptide substrates. Initial rates are plotted at indicated total SAM concentrations and the curve fit to $V = Vmax*[S]/(K_M + [S])$. RIGHT: The initial rates of methylation by G9a-GLP on $H3_{1\text{-}20}$ peptide were re-measured independently with different concentrations of cold SAM, while keeping ^{3}H-SAM constant at 9μM. **B.** Initial rates of methylation by G9a-GLP on mononucloesomes (5μM) was measured with either only ^{3}H-SAM (10μM) or 10μM ^{3}H-SAM plus 40μM cold SAM. **C.** Example Tritium screen exposures used for the Michaelis-Menten curve in Figure 3 E (G9a-GLP). **D.** Kinetic parameters of G9a-GLP methylation of $H3_{1\text{-}20}$ peptides under 100mM or 0mM (no salt) KCl. Note the drop in K_M under no salt. Error bars in A. and B. indicate standard deviation of two repeats.

Supporting Figure 4: Role of K27 in nucleosome methylation by G9a-GLP. G9a-GLPS, G9a, and G9a-GLP SET domain methylation kinetics. A. Estimation of methylation at H3K9 vs. H3K27 by G9a-GLP on mononucleosomes. Methylation was conducted under Single Turnover conditions, with enzyme>>substrate (20μM G9a-GLP, 0.16μM WT or K27A mononucleosome). Methylation time courses were fit to $P(t)=A_0*(1-e^{-kt})$ where k is the k_{obs} of the reaction. Solving for A_0 yields the number of total methylation events, and given the known [mononucleosome], allows estimation of methylation events/nucleosome. **B.** A magnification of Michaels-Menten fit for G9a-G9a Multiple Turnover kinetics as shown in Figure 3E. **C.** Initial rate measurements for G9a and G9a-GLPS under saturating concentration (V_{max}) of $H3_{1\text{-}20}$ (300μM). The estimated maximal rate is only marginally different. **D.** Michaelis-Menten multiple turnover curve for G9a-GLP SET domains with $H3_{1\text{-}20}$ peptides. The K_M is not lower than ANK-SET versions. All error bars indicate standard deviation of two replicates.

Supporting Figure 5: The SET domain active site of G9a is required for nucleosome binding. Fluorescence polarization of mononucleosomes containing 147bp 601 DNA 5' fluorescein-labeled bound to **A.** catalytically inactive G9aS-G9aS and **B.** G9aS-GLP. Error bars indicate standard deviation of two replicates.

Supporting Figure 6: G9a-GLP does not bind DNA. A. G9a-GLP heterodimer or the monomeric fission yeast Clr4 H3K9 methyltransferase were assessed for binding the fluorescein-labeled 147bp '601' mononucleosome DNA template. **B.** Electro-Mobility Shift Assay with G9a-GLP or Clr4 at indicated concentrations. **C.** Fluorescence polarization assay with G9a-GLP or Clr4 at indicated concentrations. Error bars indicate standard deviation of two replicates.

Supporting Figure 7: Crosslinking Mass Spectrometry for tagged G9a, GLP, and G9a-GLP. Crosslinking Mass Spectrometry for MBP:G9a (**A**), His:G9a-MBP:GLP (**B**), and MBP:GLP (**C**). Dot size and color indicate number of crosslinked spectral matches (CSM) summarized for domain pairs. Note that G9a and GLP data by necessity include intra-and inter protomer crosslinks. Note crosslinks involving the MBP tag.

Supporting Figure 8: Domain annotations of tagged and tag-cleaved G9a and GLP proteins Page 1: Amino acid sequence of the tag-cleaved G9a and GLP proteins. Domain identifiers are based on Uniprot and used in Figure 4 D-F and SFigure 7 A-C. Page 2: Annotated amino acid sequence of the tagged G9a and GLP proteins.

Supporting Table 1: Crosslinking Mass Spectrometry data table. Source data for the plots in Figure 4 D.-F. Q96KQ7, Uniport ID for G9a, Q9H9B1; Uniprot ID for GLP. Domain identifiers as in SFigure 8.

Supporting Figure 1

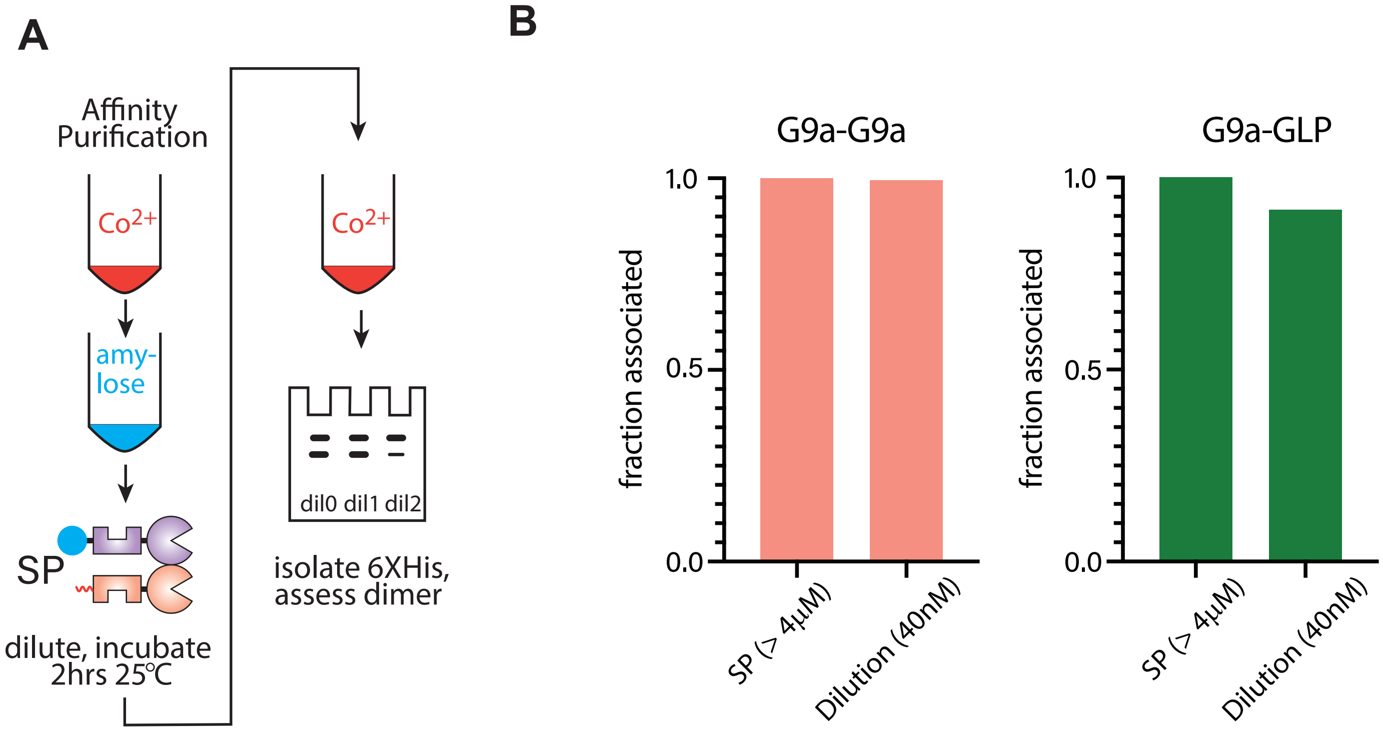

Supporting Figure 2

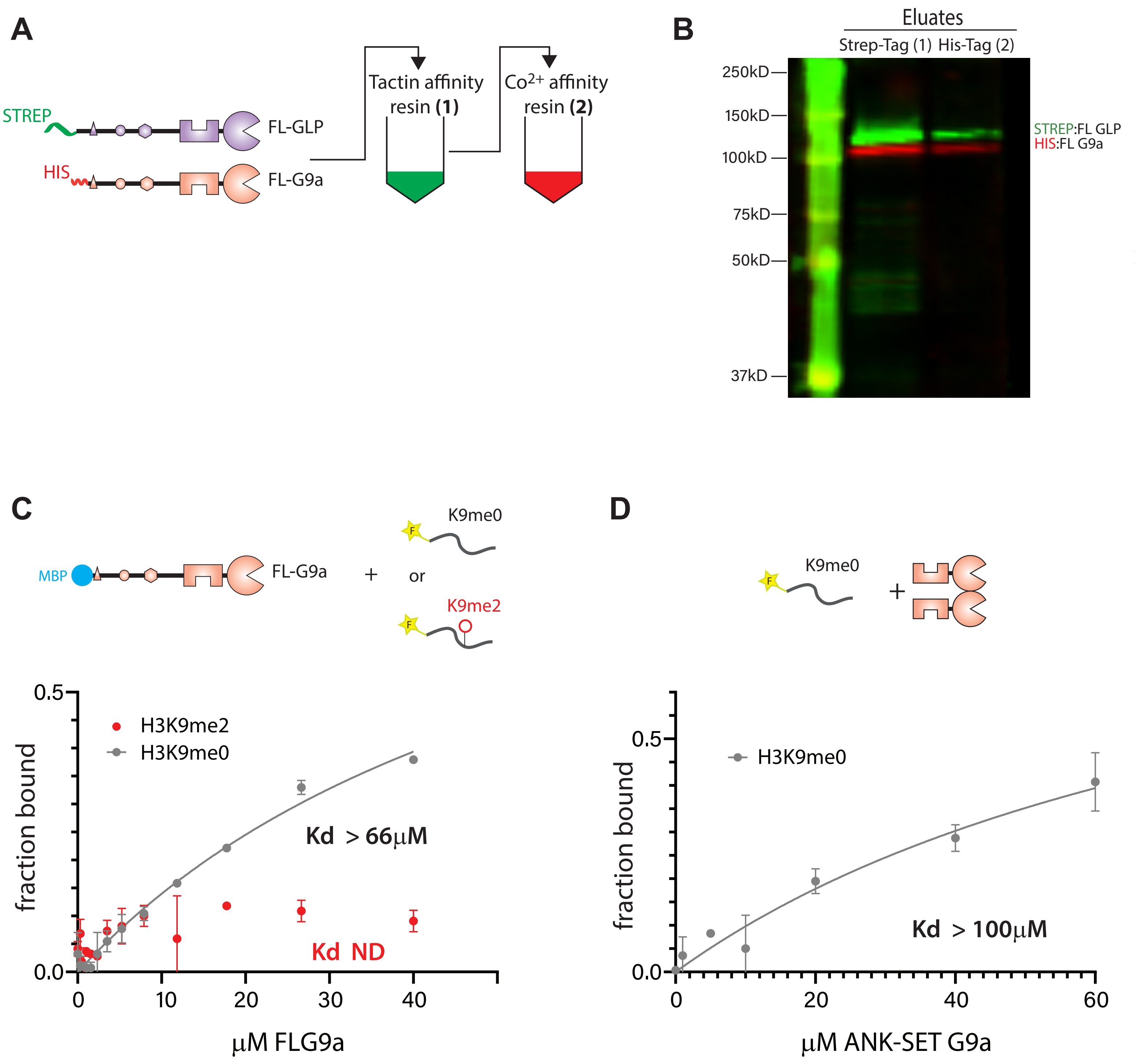

Supporting Figure 3

A

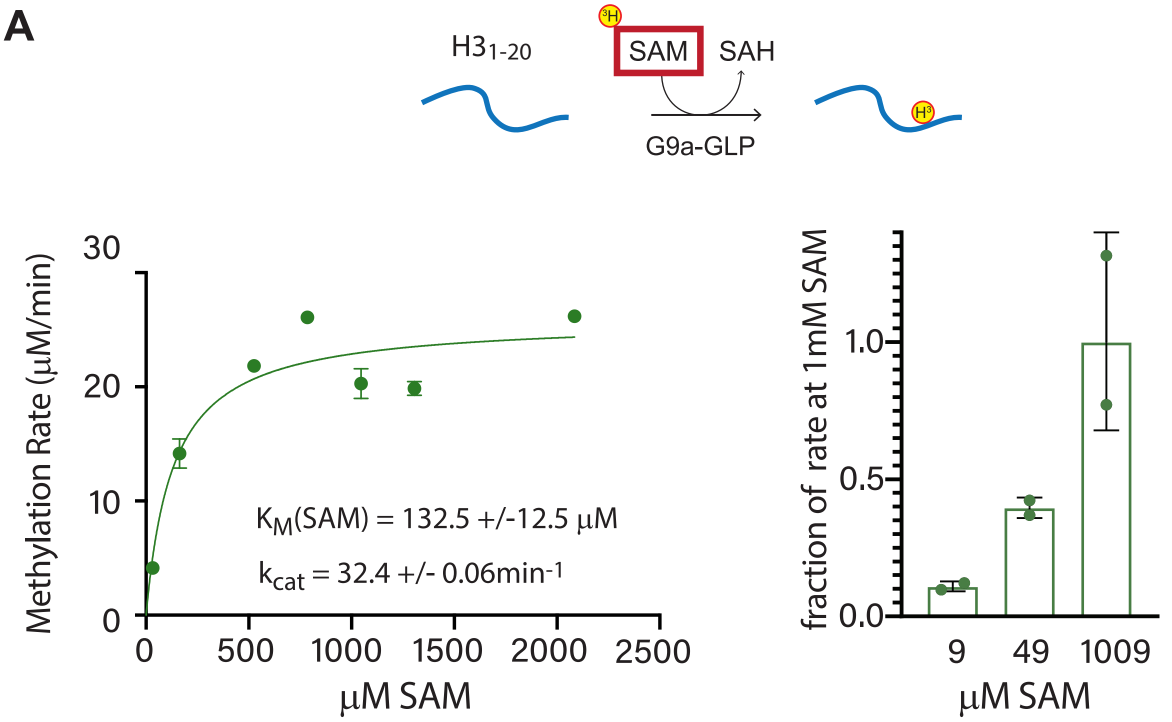

B

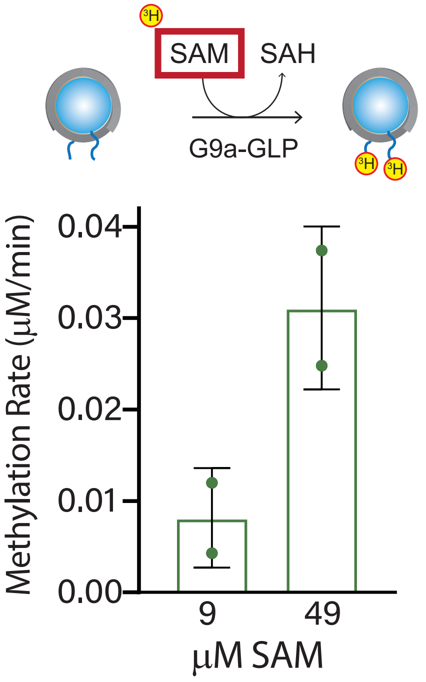

C

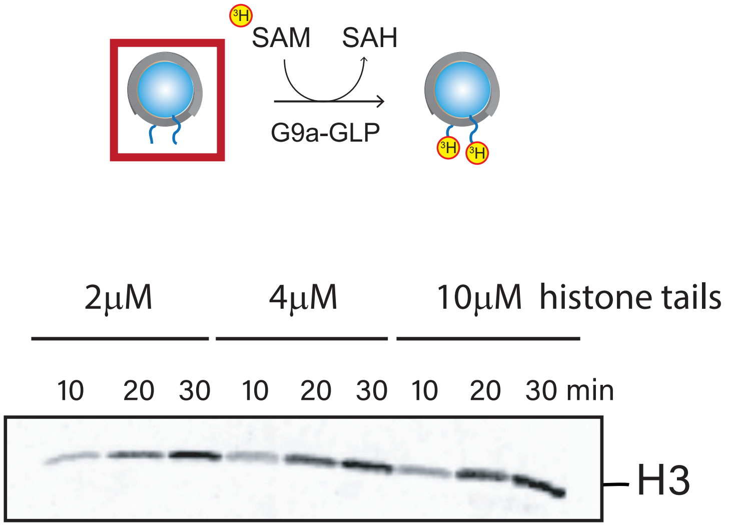

D

[KCl]	H3$_{1-20}$ peptide v_{max} (μMmin^{-1})	K_M (μM)
100mM	6.3	**120.5**
0mM	7.6	**19.5**

Supporting Figure 4

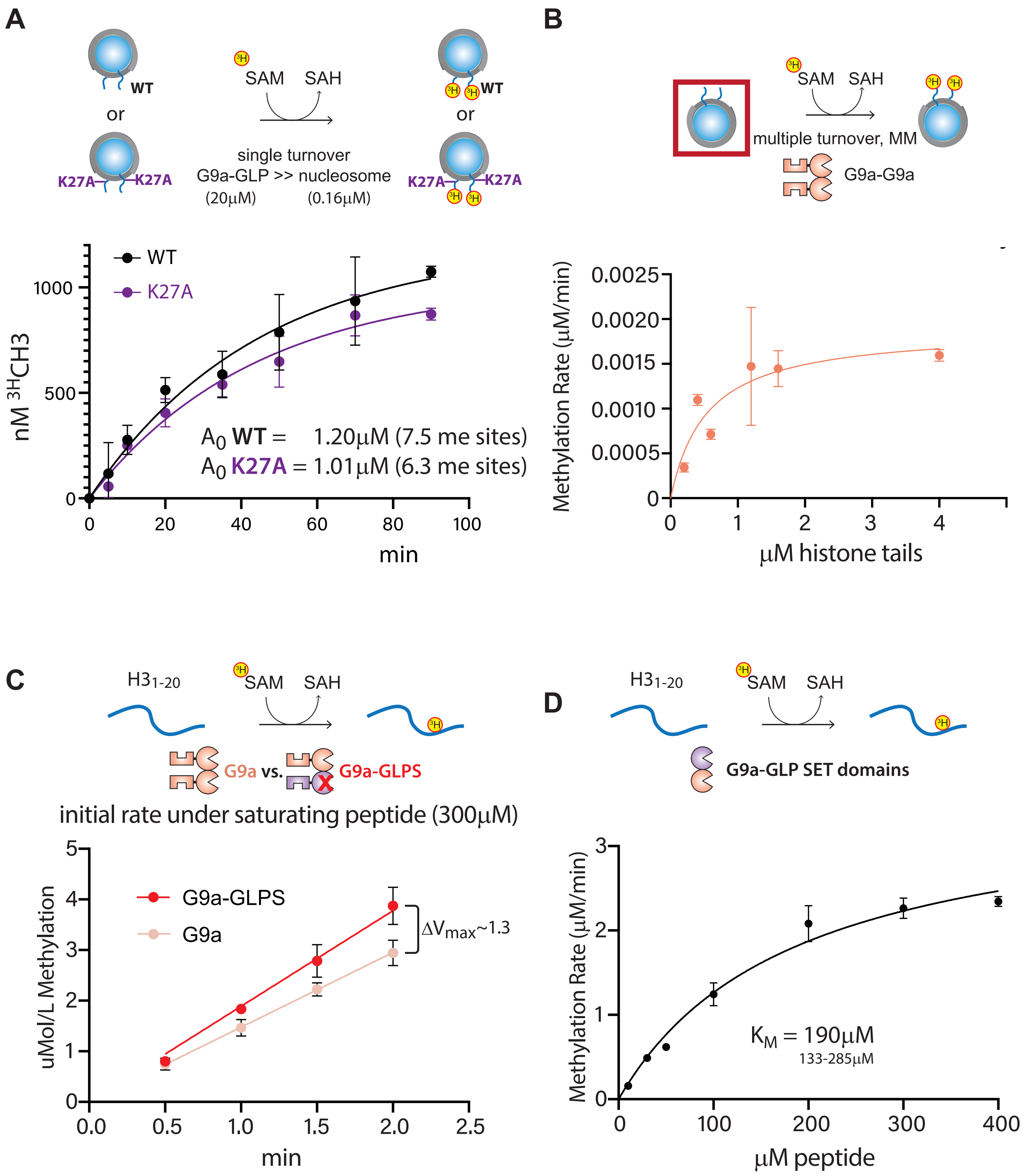

Supporting Figure 5

A

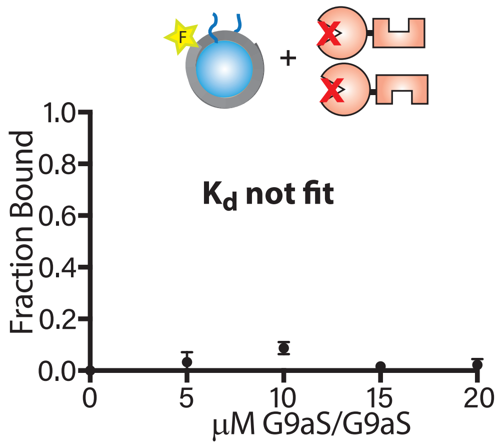

B

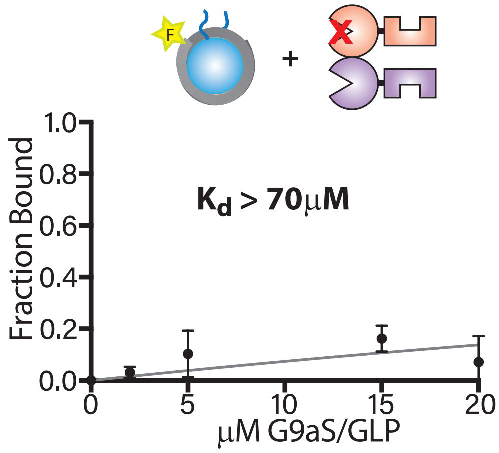

Supporting Figure 6

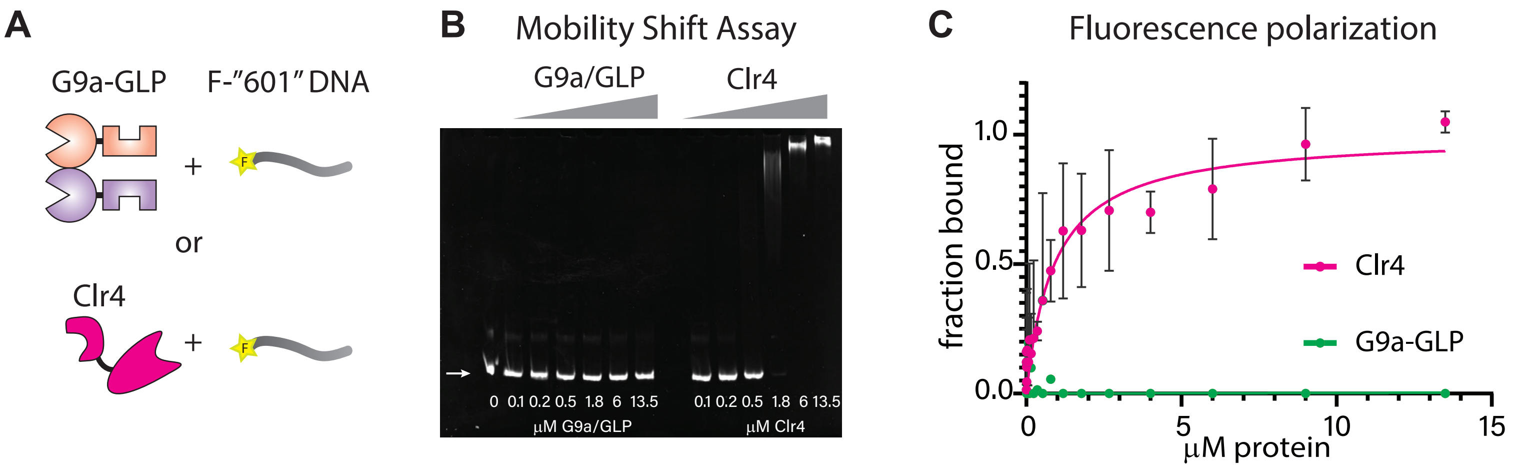

Supporting Figure 7

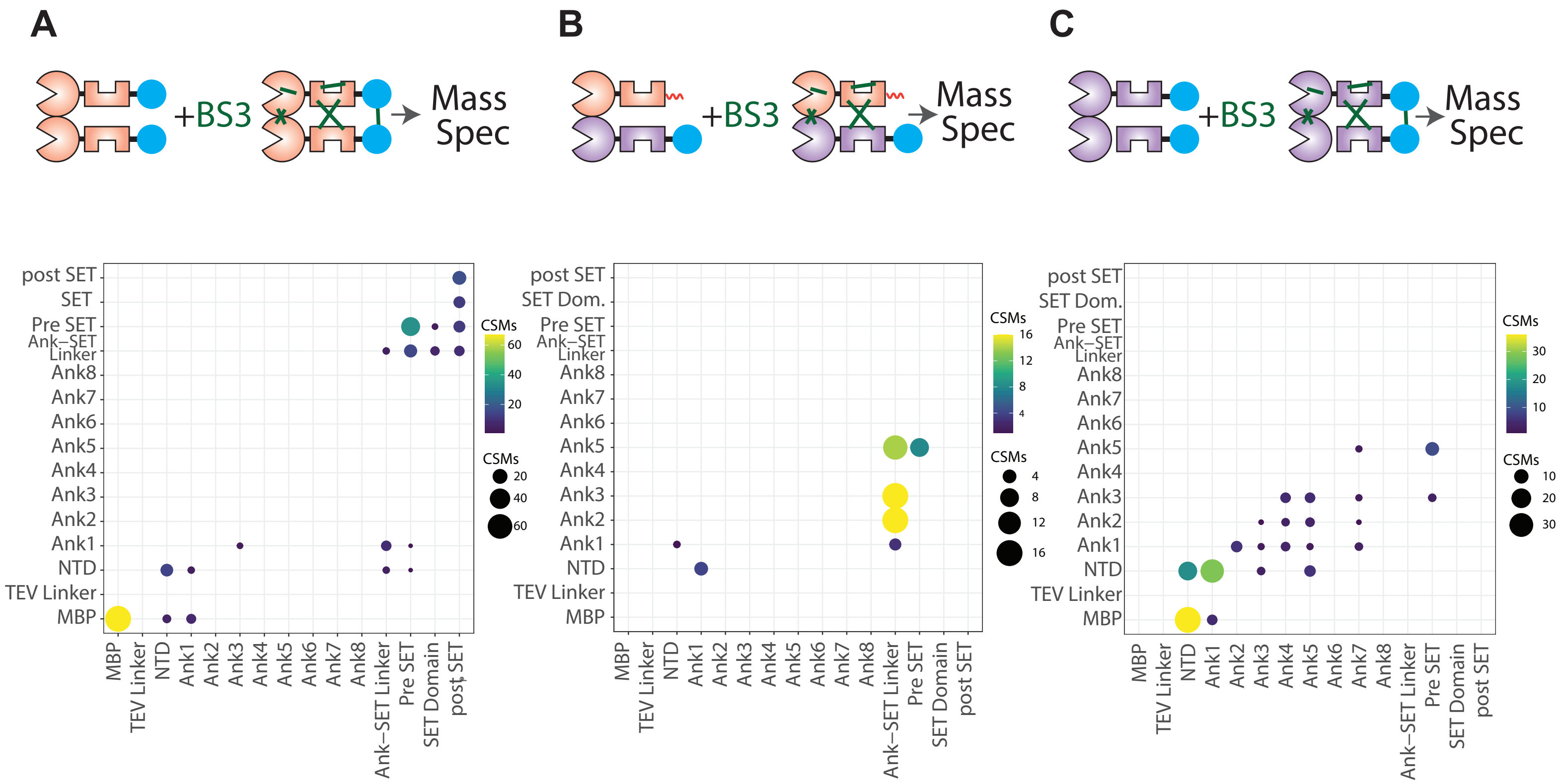

Supporting Figure 8

Cleaved Protein Sequences

G9a

SNADTIDSSGPSLTLPNGGCLSAVGLPLGPGREALEKALVIQESERRKKLRFHPRQLYLSVKQGELQKVILMLLDNLDPNFQSDQQSKRTPLHA
AAQKGSVEICHVLLQAGANINAVDKQQRTPLMEAVVNNHLEVARYMVQRGGCVYSKEEDGSTCLHHAAKIGNLEMVSLLLSTGQVDVNA
QDSGGWTPIIWAAEHKHIEVIRMLLTRGADVTLTDNEENICLHWASFTGSAAIAEVLLNARCDLHAVNYHGDTPLHIAARESYHDCVLLFLSR
GANPELRNKEGDTAWDLTPERSDVWFALQLNRKLRLGVGNRAIRTEKIICRDVARGYENVPIPCVNGVDGEPCPEDYKYISENCETSTMNID
RNITHLQHCTCVDDCSSSNCLCGQLSIRCWYDKDGRLLQEFNKIEPPLIFECNQACSCWRNCKNRVVQSGIKVRLQLYRTAKMGWGVRALQ
TIPQGTFICEYVGELISDAEADVREDDSYLFDLDNKDGEVYCIDARYYGNISRFINHLCDPNIIPVRVFMLHQDLRFPRIAFFSSRDIRTGEELGFD
YGDRFWDIKSKYFTCQCGSEKCKHSAEAIALEQSRLARLDPHPELLPELGSLPPVNT

N-Terminus Ank 1 Ank 2 Ank 3 Ank 4 Ank 5 Ank 6 Ank 7 ANK-SET Linker Pre SET Domain SET Domain Post SET Domain

GLP

SNASKAKEVTIAKADTTSTVTPVPGQEKGSALEGRADTTTGSAAGPPLSEDDKLQGAASHVPEGFDPTGPAGLGRPTPGLSQGPGKETLESAL
IALDSEKPKKLRFHPKQLYFSARQGELQKVLLMLVDGIDPNFKMEHQNKRSPLHAAAEAGHVDICHMLVQAGANIDTCSEDQRTPLMEAAE
NNHLEAVKYLIKAGALVDPKDAEGSTCLHLAAKKGHYEVVQYLLSNGQMDVNCQDDGGWTPMIWATEYKHVDLVKLLLSKGSDINIRDNE
ENICLHWAAFSGCVDIAEILLAAKCDLHAVNIHGDSPLHIAARENRYDCVVLFLSRDSDVTLKNKEGETPLQCASLNSQVWSALQMSKALQDS
APDRPSPVERIVSRDIARGYERIPIPCVNAVDSEPCPSNYKYVSQNCVTSPMNIDRNITHLQYCVCIDDCSSSNCMCGQLSMRCWYDKDGRLL
PEFNMAEPPLIFECNHACSCWRNCRNRVVQNGLRARLQLYRTRDMGWGVRSLQDIPPGTFVCEYVGELISDSEADVREEDSYLFDLDNKDG
EVYCIDARFYGNVSRFINHHCEPNLVPVRVFMAHQDLRFPRIAFFSTRLIEAGEQLGFDYGERFWDIKGKLFSCRCGSPKCRHSSAALAQRQA
SAAQEAQEDGLPDTSSAAAADPL

N-Terminus Ank 1 Ank 2 Ank 3 Ank4 Ank5 Ank6 Ank 7 Ank 8 Ank-SET Linker Pre SET SET Domain Post SET Domain

Uncleaved Protein Sequences

MBP-G9a

MGSSGSKIEEGKLVIWINGDKGYNGLAEVGKKFEKDTGIKVTVEHPDKLEEKFPQVAATGDGPDIIFWAHDRFGGYAQSGLLAEITPDKAFQ
DKLYPFTWDAVRYNGKLIAYPIAVEALSLIYNKDLLPNPPKTWEEIPALDKELKAKGKSALMFNLQEPYFTWPLIAADGGYAFKYENGKYDIKD
VGVDNAGAKAGLTFLVDLIKNKHMNADTDYSIAEAAFNKGETAMTINGPWAWSNIDTSKVNYGVTVLPTFKGQPSKPFVGVLSAGINAASP
NKELAKEFLENYLLTDEGLEAVNKDKPLGAVALKSYEEELAKDPRIAATMENAQKGEIMPNIPQMSAFWYAVRTAVINAASGRQTVDEALKD
AQTNENLYFQSNADTIDSSGPSLTLPNGGCLSAVGLPLGPGREALEKALVIQESERRKKLRFHPRQLYLSVKQGELQKVILMLLDNLDPNFQSD
QQSKRTPLHAAAQKGSVEICHVLLQAGANINAVDKQQRTPLMEAVVNNHLEVARYMVQRGGCVYSKEEDGSTCLHHAAKIGNLEMVSLLL
STGQVDVNAQDSGGWTPIIWAAEHKHIEVIRMLLTRGADVTLTDNEENICLHWASFTGSAIAEVLLNARCDLHAVNYHGDTPLHIAARESY
HDCVLLFLSRGANPELRNKEGDTAWDLTPERSDVWFALQLNRKLRLGVGNRAIRTEKIICRDVARGYENVPIPCVNGVDGEPCPEDYKYISEN
CETSTMNIDRNITHLQ

HCTCVDDCSSSNCLCGQLSIRCWYDKDGRLLQEFNKIEPPLIFECNQACSCWRNCKNRVVQSGIKVRLQLYRTAKMGWGVRALQTIPQGTFI
CEYVGELISDAEADVREDDSYLFDLDNKDGEVYCIDARYYGNISRFINHLCDPNIIPVRVFMLHQDLRFPRIAFFSSRDIRTGEELGFDYGDRFW
DIKSKYFTCQCGSEKCKHSAEAIALEQSRLARLDPHPELLPELGSLPPVNT

MBP TEV Cleavage Site N-Terminus Ank 1 Ank 2 Ank 3 Ank 4 Ank 5 Ank 6 Ank 7 ANK-SET Linker Pre SET Domain SET Domain Post SET Domain

MBP-GLP

MGSSGSKIEEGKLVIWINGDKGYNGLAEVGKKFEKDTGIKVTVEHPDKLEEKFPQVAATGDGPDIIFWAHDRFGGYAQSGLLAEITPDKAFQ
DKLYPFTWDAVRYNGKLIAYPIAVEALSLIYNKDLLPNPPKTWEEIPALDKELKAKGKSALMFNLQEPYFTWPLIAADGGYAFKYENGKYDIKD
VGVDNAGAKAGLTFLVDLIKNKHMNADTDYSIAEAAFNKGETAMTINGPWAWSNIDTSKVNYGVTVLPTFKGQPSKPFVGVLSAGINAASP
NKELAKEFLENYLLTDEGLEAVNKDKPLGAVALKSYEEELAKDPRIAATMENAQKGEIMPNIPQMSAFWYAVRTAVINAASGRQTVDEALKD
AQTNENLYFQSNASKAKEVTIAKADTTSTVTPVPGQEKGSALEGRADTTTGSAAGPPLSEDDKLQGAASHVPEGFDPTGPAGLGRPTPGLSQ
GPGKETLESALIALDSEKPKKLRFHPKQLYFSARQGELQKVLLMLVDGIDPNFKMEHQNKRSPLHAAAEAGHVDICHMLVQAGANIDTCSED
QRTPLMEAAENNHLEAVKYLIKAGALVDPKDAEGSTCLHLAAKKGHYEVVQYLLSNGQMDVNCQDDGGWTPMIWATEYKHVDLVKLLLS
KGSDINIRDNEENICLHWAAFSGCVDIAEILLAAKCDLHAVNIHGDSPLHIAARENRYDCVVLFLSRDSDVTLKNKEGETPLQCASLNSQVWSA
LQMSKALQDSAPDRPSPVERIVSRDIARGYERIPIPCVNAVDSEPCPSNYKYVSQNCVTSPMNIDRNITHLQYCVCIDDCSSSNCMCGQLSMR
CWYDKDGRLLPEFNMAEPPLIFECNHACSCWRNCRNRVVQNGLRARLQLYRTRDMGWGVRSLQDIPPGTFVCEYVGELISDSEADVREED
SYLFDLDNKDGEVYCIDARFYGNVSRFINHHCEPNLVPVRVFMAHQDLRFPRIAFFSTRLIEAGEQLGFDYGERFWDIKGKLFSCRCGSPKCRH
SSAALAQRQASAAQEAQEDGLPDTSSAAAADPL

MBP TEV Cleavage Site N-Terminus Ank 1 Ank 2 Ank 3 Ank4 Ank5 Ank6 Ank 7 Ank 8 Ank-SET Linker Pre SET SET Domain Post SET Domain

HIS-G9a

MGSSHHHHHHENLYFQSNADTIDSSGPSLTLPNGGCLSAVGLPLGPGREALEKALVIQESERRKKLRFHPRQLYLSVKQGELQKVILMLLDNL
DPNFQSDQQSKRTPLHAAAQKGSVEICHVLLQAGANINAVDKQQRTPLMEAVVNNHLEVARYMVQRGGCVYSKEEDGSTCLHHAAKIGN
LEMVSLLLSTGQVDVNAQDSGGWTPIIWAAEHKHIEVIRMLLTRGADVTLTDNEENICLHWASFTGSAIAEVLLNARCDLHAVNYHGDTPL
HIAARESYHDCVLLFLSRGANPELRNKEGDTAWDLTPERSDVWFALQLNRKLRLGVGNRAIRTEKIICRDVARGYENVPIPCVNGVDGEPCPE
DYKYISENCETSTMNIDRNITHLQHCTCVDDCSSSNCLCGQLSIRCWYDKDGRLLQEFNKIEPPLIFECNQACSCWRNCKNRVVQSGIKVRLQ
LYRTAKMGWGVRALQTIPQGTFICEYVGELISDAEADVREDDSYLFDLDNKDGEVYCIDARYYGNISRFINHLCDPNIIPVRVFMLHQDLRFPR
IAFFSSRDIRTGEELGFDYGDRFWDIKSKYFTCQCGSEKCKHSAEAIALEQSRLARLDPHPELLPELGSLPPVNT

MBP TEV Cleavage Site N-Terminus Ank 1 Ank 2 Ank 3 Ank 4 Ank 5 Ank 6 Ank 7 ANK-SET Linker Pre SET Domain SET Domain Post SET Domain
